# Chestnut (*Castanea crenata*) Inner-Shell Extract Attenuates Barium-Chloride-Induced Injury and Denervation-Induced Atrophy in Skeletal Muscle of Mice

**DOI:** 10.3390/nu17132116

**Published:** 2025-06-26

**Authors:** Jin-Hwa Kim, Eun-Hye Chung, Jeong-Won Kim, Ji-Soo Jeong, Chang-Yeop Kim, Su-Ha Lee, Je-Won Ko, Je-Oh Lim, Tae-Won Kim

**Affiliations:** 1 BK21 FOUR Program, College of Veterinary Medicine, Chungnam National University, 99 Daehak-ro, Daejeon 34131, Republic of Korea; jinhwa926@g.cnu.ac.kr (J.-H.K.); ksissb1293@gmail.com (E.-H.C.); lilflflb@gmail.com (J.-W.K.); jisooj9543@gmail.com (J.-S.J.); 963ckdduq@gmail.com (C.-Y.K.); suhai2729@gmail.com (S.-H.L.); rheoda@cnu.ac.kr (J.-W.K.); 2 Laboratory of Radiation Exposure & Therapeutics, National Radiation Emergency Medical Center, Korea Institute of Radiological and Medical Sciences, Seoul 01812, Republic of Korea; 3 Division of Jeonbuk Advanced Bio Research, Korea Institute of Toxicology, Jeongeup 53212, Republic of Korea; 4 Herbal Medicine Resources Research Center, Korea Institute of Oriental Medicine, Naju 58245, Republic of Korea

**Keywords:** barium chloride, chestnut inner shell, denervation, oxidative stress, skeletal muscle disorders

## Abstract

**Background/Objectives**: Chestnut inner shells, traditionally used in Korean and Chinese herbal medicine, contain antioxidant and anti-inflammatory compounds that contribute to complementary medicine. This study aimed to explore the therapeutic effects of chestnut inner-shell extract (CIE) on skeletal muscle injury and atrophy using both in vivo and in vitro models. **Methods**: We used three experimental models representing distinct pathological mechanisms: (1) barium chloride (BaCl_2_)-induced muscle injury to model acute myofiber damage, (2) sciatic nerve transection to model chronic neurogenic muscle atrophy, and (3) H_2_O_2_-treated C2C12 myoblasts to model oxidative-stress-related myogenic impairment. Histological analyses (e.g., hematoxylin and eosin staining and cross-sectional area measurement) and molecular analyses were performed to evaluate the effects of CIE on muscle structure, apoptosis, and oxidative stress. **Results**: In the BaCl_2_ injury model, CIE treatment significantly restored the muscle fiber structure, with muscle protein levels returning to near-normal levels. In the denervation-induced muscle atrophy model, CIE treatment led to a dose-dependent decrease in apoptosis-related factors (especially cleaved caspase-3) and mitigated the Akt/mTOR signaling pathway. In the in vitro oxidative stress model, CIE suppressed the expression of NRF2 and HO-1, which are key oxidative stress response regulators. **Conclusions**: These findings suggest that CIE may offer therapeutic potential for mitigating skeletal muscle damage, atrophy, and oxidative stress.

## 1. Introduction

Skeletal muscle disorders arise from various causes, resulting in the considerable impairment of muscle function and general health [1]. Denervation and chemical-injection injury are standard experimental models associated with muscle disorders [2]. The barium chloride (BaCl_2_) injection model causes acute muscle damage, making it crucial for investigating muscle regeneration [3]. BaCl_2_ induces necrosis primarily through mechanisms involving calcium (Ca^2+^) overload and subsequent cellular damage [4]. Elevated Ca^2+^ levels can lead to mitochondrial dysfunction, a common feature in necrotic cell death [5]. The sciatic nerve transection model is utilized to examine muscle loss caused by nerve damage, which results in atrophy [6] and is further aggravated by oxidative stress and inflammatory responses [7]. Reactive oxygen species (ROS) inhibits the differentiation of myoblasts, promotes muscle atrophy through the breakdown of differentiated muscle cells, and functions as a pathological factor in several muscle disorders [8]. H_2_O_2_ is utilized to investigate oxidative-stress-related damage in both myoblasts and myotubes [9].

Due to increased mortality and complication rates linked to skeletal muscle disorders in various clinical cases, there is a rising demand for effective and sustainable treatment options. Recent clinical trials have begun to investigate phytotherapeutic agents for musculoskeletal disorders, including herbal formulations targeting sarcopenia and muscle dystrophy [10,11]. The application of phytotherapy for managing muscle disorders is gaining traction due to its safety profile and accessibility [12]. Chestnut (*Castanea crenata*) has been traditionally used as a medicinal herb in Asia. The antioxidant and anti-inflammatory properties are the pharmacological benefits of the chestnut [13]. Earlier investigations indicated that extracts from chestnut flour positively affect muscle atrophy modulation by reducing myotube size and protein degradation in C2C12 myoblasts [14]. Additionally, chestnut inner-shell extract (CIE) offers potential benefits due to its elevated levels of phenolic compounds, such as tannic, gallic, and ellagic acids, compared to other parts of the chestnut [15]. Theses phenolic acids have been reported to exert muscle-protective effects by alleviating oxidative stress and inflammation [16,17]. Nevertheless, the effects of CIE on skeletal muscle damage and atrophy remain underexplored.

This study aimed to assess the efficacy of CIE in reducing acute muscle injuries and addressing muscle atrophy caused by denervation. Two in vivo models were utilized: the BaCl_2_ injection model for muscle damage and the sciatic nerve transection model for muscle atrophy. Additionally, we examined the effects of CIE when oxidative stress was induced in C2C12 myoblasts.

## 2. Materials and Methods

### 2.1. Chestnut Inner-Shell Extract Preparation

The CIE was prepared as previously described [13]. Dried chestnut inner shell powder was obtained from Jangmyung (Seoul, Republic of Korea). A total of 300 g of the powder was extracted in 6 L of 70% ethanol at 25 °C for 24 h. The supernatant was collected, filtered, and concentrated in a rotary evaporator at 40 °C (Heidolph, Schwabach, Germany). The extract was subsequently lyophilized, and the yield from the extraction was calculated to be 25.05%. High-performance liquid chromatography (HPLC) was conducted to measure the phytochemicals in CIE, as previously reported, and the ellagic and gallic acid contents were 0.17 and 0.04%, respectively [13].

### 2.2. Animals

Male C57BL/6 mice were obtained from SAMTAKO (Osan, Republic of Korea) and acclimatized for one week before the experiment. The mice were maintained under appropriate environmental conditions and provided standard rodent chow and water ad libitum. All animal experiments were approved by and conducted according to the guidelines of the Institutional Animal Experiments (202112A-CNU-215).

### 2.3. Establishment of BaCl_2_-Induced Muscle Injury Model and Treatment

After acclimatization for one week, thirty mice were randomly assigned to five groups (*n* = 6/group): (1) NC (normal control, phosphate-buffered saline [PBS] per oral [P.O.]); (2) BaCl_2_ (BaCl_2_ + PBS P.O.); and (3–5) CIE (BaCl_2_ injection + CIE 100, 200, and 400 mg/kg/day P.O., respectively). PBS or CIE was administered daily for five days prior to BaCl_2_ injection. All mice, except those in the NC group, received an intramuscular (IM) injection of 50 μL of 1.2% BaCl_2_ solution (dissolved in PBS) into the right tibialis anterior (TA) muscle under anesthesia induced by an isoflurane–oxygen mixture. IM injection was performed using a 1 cc syringe fitted with a 30-gauge needle. The needle was inserted to a depth sufficient to view expansion of the muscle (~2 mm). The left hindlimb remained intact. Following BaCl_2_ injection, either PBS or CIE was administered continuously once daily for ten days. The body weight and grip strength were measured before the injection of BaCl_2_ and on days 1 and 10 post-injection. Group allocation and data collection were conducted in a blinded manner.

### 2.4. Establishment of Muscle Denervation Model and Treatment

After acclimatization for one week, twenty-four mice were randomly allocated to four groups as follows (*n* = 6/group): (1) sham (sham control, PBS P.O.); (2) Den (denervation group, PBS P.O.); and (3) CIE (denervation + CIE 100 or 400 mg/kg/day P.O.). All mice except those in the sham group underwent transection of their peripheral nerve under anesthesia using Zoletil50^®^ (10 mg/kg; Virbac Laboratories, Carros, France), as previously reported [18]. The sciatic nerve was exposed via an incision along the medial side of the right thigh and excised precisely at its midline under anesthesia. The sham mice underwent a skin incision at the same position to expose the sciatic nerve, but the nerves were not touched during the surgery. After denervation, the mice were orally administered PBS or CIE daily for 30 days. The body weights of all mice were recorded once every seven days. Group assignment and data recording were performed in a blinded manner.

### 2.5. Grip Strength Test

Grip strength is a crucial index for studying muscle physiology in disease and muscle function by measuring muscle force in active laboratory mice [19]. Hind limb grip strength was measured using a computerized grip strength meter (Ugo-Basile, Varese, Italy). All measurements were performed by a single experimenter in a blinded manner to minimize bias. Mice were held on a wooden stick to prevent accidental gripping of the meter with their other legs, while the tester carefully positioned each back leg on the device by holding the base of the mouse’s tail. The mouse was gently pulled backward until it released its grip, and the peak force was automatically recorded in grams (g). Grip strength was measured six times per mouse, and the average value was used for analysis. Mice were weighed on the days of their grip strength testing to normalize peak force measurements relative to body weight (g force/g body weight).

### 2.6. Histological Analysis

Skeletal muscle samples were dehydrated, embedded in paraffin, and sectioned at a thickness of 4 μm. Histological evaluation was performed through hematoxylin and eosin (H&E) staining (TissuePro Technology, Gainesville, FL, USA). The morphology of skeletal muscles was observed using an optical microscope (Leica, Wetzlar, Germany) by an investigator blinded to the group allocation. For each section, five non-overlapping fields were randomly captured at an identical magnification. Centrally nucleated fibers were quantified in a blinded manner using ImageJ (version 1.8.0_172, National Institutes of Health, Bethesda, MD, USA) and expressed as a percentage of total fibers within the selected region. The reduction in muscle mass diameter in the denervation model was assessed via immunofluorescence (IF) using anti-laminin (1:50; Abcam, Cambridge, UK). The cross-sectional area (CSA) of muscle fibers was quantified using ImageJ software. Myogenin (MyoG) expression in TA muscles was evaluated via immunohistochemistry (IHC) using a primary antibody against MyoG (1:500; Abcam). IF and IHC staining were performed as described previously [18]. The quantitative analysis of stained sections was conducted using ImageJ software (version 1.8.0_172) by a blinded evaluator.

### 2.7. Terminal Deoxynucleotidyl Transferase dUTP Nick End Labeling Assay

To evaluate the impact of CIE on apoptosis induced by denervation in TA [20], we conducted a terminal deoxynucleotidyl transferase dUTP nick end labeling (TUNEL) assay using a kit from Abcam, following the manufacturer’s guidelines. After counterstaining with hematoxylin, the specimens were mounted on coverslips. Quantification was conducted using ImageJ software by a researcher who was blinded to group assignments.

### 2.8. Immunoblotting

The total protein concentration was determined using the Pierce™ BCA Protein Assay (Thermo Fisher Scientific, Waltham, MA, USA), and 20 μg of protein per sample was loaded onto polyacrylamide gels, followed by transfer to PVDF membranes (Millipore, Burlington, MA, USA). The membranes were blocked with 5% bovine serum albumin in 0.1% Tween 20 in PBS for 1 h and then incubated overnight at 4 °C with primary antibodies. The primary antibodies used included the following: myostatin (1:1000; Abcam), myogenic differentiation 1 (MyoD; 1:1000; Abcam), MyoG (1:1000; Abcam), muscle RING-finger protein-1 (MuRF1; 1:1000; Abcam), total (t)-protein kinase B (t-AKT; 1:1000; Abcam), p-AKT (1:1000; Abcam), t-mammalian target of rapamycin (t-mTOR; 1:1000; GeneTex, Irvine, CA, USA), p-mTOR (1:1000; GeneTex), Bcl-2-associated X protein (Bax; 1:1000; Abcam), cytochrome C (1:1000; Abcam), cleaved caspase-3 (1:1000; Cell Signaling Technology, Danvers, MA, USA), nuclear factor erythroid 2-related factor 2 (NRF2; 1:1000; Abcam), and heme oxygenase 1 (HO1; 1:1000; Abcam). After washing, the membranes were incubated with the corresponding secondary antibodies for 2 h at 24 °C. Band intensities were visualized using a ChemiDoc imaging system (Bio-Rad Laboratories, Hercules, CA, USA), and quantification was conducted using ImageJ software by an investigator blinded to the group assignments. Ponceau S staining was used to assess total protein levels for normalization. Given the variability of commonly used housekeeping proteins (e.g., GAPDH, β-actin, tubulin) in muscle tissues due to fiber-type differences and pathological states, Ponceau S staining was used as an alternative loading control [21,22].

### 2.9. Cell Culture

C2C12 myoblasts were obtained from the American Type Culture Collection (ATCC; Manassas, VA, USA) and cultured in Dulbecco’s Modified Eagle Medium (DMEM; Sigma-Aldrich, St. Louis, MO, USA) supplemented with 10% fetal bovine serum (Gibco, Carlsbad, CA, USA) and 1% penicillin–streptomycin (Sigma-Aldrich) at 37 °C in a humidified atmosphere at 5% CO_2_.

### 2.10. Cell Viability Assay

C2C12 myoblasts were seeded in 96-well plates at a density of 5 × 10^4^ cells/mL and incubated overnight. The cells were then treated with either vehicle (0.1% DMSO) or CIE at concentrations ranging from 0 to 80 μg/mL using two-fold serial dilutions in growth medium for 24 h. Cell viability was assessed using EZ-Cytox solution (DoGenBio Co., Seoul, Republic of Korea) by adding 10 μL of the reagent per well and incubating for 1 h. Absorbance was measured at 450 nm using a microplate reader (Bio-Rad Laboratories). To evaluate oxidative-stress-induced cytotoxicity, cells were treated with hydrogen peroxide (H_2_O_2_) at concentrations ranging from 0 to 1 mM (0.2 mM intervals). To assess the protective effect of CIE, cells were pre-treated with vehicle or CIE for 1 h prior to H_2_O_2_ exposure for an additional 1 h. N-acetyl-L-cysteine (NAC; 2 mM in PBS; Sigma-Aldrich) was used as a positive control.

### 2.11. Statistical Analyses

The results are expressed as the mean ± standard deviation (SD). Data normality was assessed using the Shapiro–Wilk test. For normally distributed data, statistical differences among groups were analyzed using one-way analysis of variance (ANOVA), followed by Tukey’s post-hoc test. For non-normally distributed data, the Kruskal–Wallis test was applied. Statistical significance was set at *p* < 0.05. Statistical significance was determined using GraphPad InStat (version 3.0; GraphPad, La Jolla, CA, USA).

## 3. Results

### 3.1. CIE Improves the TA Muscle Mass in Two Different Models

We examined how CIE affects skeletal muscle damage and atrophy using two different methods: BaCl_2_-induced muscle injury and denervation-induced muscle atrophy. Unlike the healthy TA muscle, both BaCl_2_ injection and denervation led to a notable reduction in muscle weight (^#^ *p* < 0.05, 30.9%; ^##^ *p* < 0.01, 35.0%) (Table 1 and Table 2). No significant results were found, but there was a trend showing improvement when comparing the CIE concentration to the BaCl_2_ group in the BaCl_2_ injury model for the ratio of injured TA muscle weight to intact TA muscle weight. Four weeks after the sciatic nerve was cut in the denervation-induced muscle wasting model, high-dose CIE treatment significantly improved the muscle weight ratio (operated/non-operated) compared to the Den group (* *p* < 0.05, 14.8%).

### 3.2. CIE Ameliorates Skeletal Muscle Function After the BaCl_2_-Induced Muscle Damage

**Figure 1:**
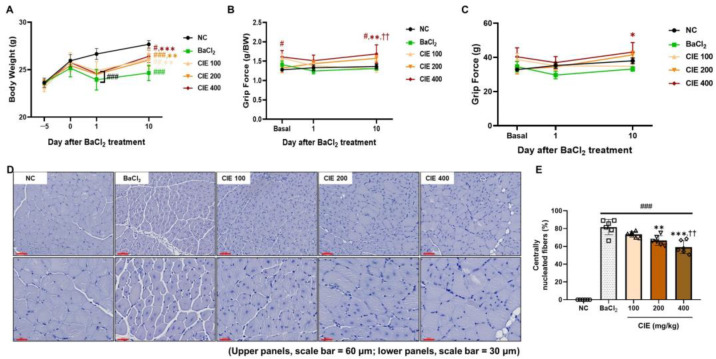
Effects of chestnut inner shell extract (CIE) treatment on the functional and histological changes in barium chloride (BaCl_2_)-induced muscle injury mouse model. (**A**) Changes in body weight were observed for 10 days following BaCl_2_ administration in different groups. (**B**) Grip strength normalized to body weight (g/BW) was evaluated at baseline and on days 1 and 10 after BaCl_2_ injection. (**C**) Absolute grip force (g) values. (**D**) Representative hematoxylin and eosin staining images of TA muscle sections from each experimental group at 200× (upper panels, scale bar = 60 μm) and 400× magnification (lower panels, scale bar = 30 μm). (**E**) Assessment of centrally nucleated fibers (%) in tibialis anterior (TA) muscle sections among all experimental groups. Data are presented as the mean ± SD. ^#^ *p* < 0.05, ^##^ *p* < 0.01, and ^###^ *p* < 0.001 vs. NC group; * *p* < 0.05, ** *p* < 0.01, and *** *p* < 0.001, vs. BaCl_2_ group; ^††^ *p* < 0.01 vs. CIE 100 group. Normal control (NC; phosphate-buffered-saline-treated group); BaCl_2_ (BaCl_2_ + PBS P.O.); and CIE (BaCl_2_ injection + CIE 100, 200, and 400 mg/kg/day P.O., respectively).

On the first day after BaCl_2_ was injected, all BaCl_2_-treated groups lost weight. However, the CIE treatment group showed greater weight recovery than the BaCl_2_ group on day 10 (Figure 1A). Grip strength tests were performed before injection and on days 1 and 10 post-injection. Compared to the NC group, the BaCl_2_ injection caused a significant drop in grip strength on day 1 after injection (*^###^ p* < 0.001, 10.2%) (Figure 1B). The CIE 400 group demonstrated the highest grip strength (g/BW) values throughout the experimental period, showing a 1.2-fold increase compared to NC (^##^ *p* < 0.01) and a 1.3-fold increase relative to both the BaCl_2_ and CIE100 groups (** *p* < 0.01 and ^††^ *p* < 0.01) on day 10 after injury. On day 10 after injury, the absolute grip force (g) in the 400 mg/kg of CIE was significantly higher than that in the BaCl_2_ group (* *p* < 0.05) (Figure 1C). The histological features of the TA muscle were analyzed on day 10 after the BaCl_2_-induced injury (Figure 1D). The group that received BaCl_2_ showed a much higher ratio of centrally nucleated myofibers than the NC group (Figure 1E; ^###^ *p* < 0.001, 81.5%). Centrally nucleated fibers are important signs of muscle regeneration and are seen after an injury [23]. The amount of centrally located nuclei in the myofibers significantly decreased in a dose-dependent way after CIE treatment (** *p* < 0.01 and *** *p* < 0.001), and in the CIE400 group, muscle morphology appeared to be restored, closely resembling that of the NC group.

### 3.3. CIE Modulates MyoD, MyoG, and Myostatin Levels in TA Muscle After the BaCl_2_-Induced Muscle Damage

**Figure 2:**
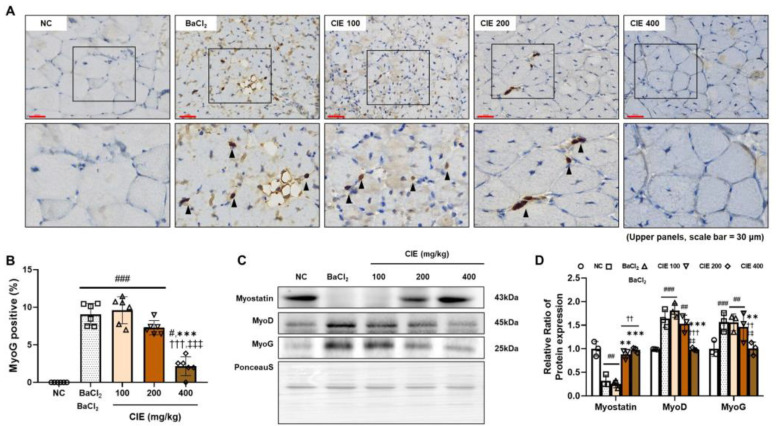
Effects of chestnut inner shell extract (CIE) on muscle regeneration in the tibialis anterior (TA) muscle following barium chloride (BaCl_2_)-induced injury. (**A**) Immunohistochemical staining of myogenin (MyoG) expression in TA muscle sections across different treatment groups. The upper panels present the entire field of view at 400× magnification (scale bar = 30 μm), while the lower panels display magnified regions (marked by boxes). MyoG-positive cells are indicated by black arrowheads. (**B**) Quantification of MyoG-positive cells per field in TA muscle sections from different treatment groups. (**C**) Western blot analysis and quantification of myostatin, myogenic differentiation 1 (MyoD), and MyoG protein levels in TA muscle tissue from different treatment groups. Full-length blots/gels are presented in Figure S1. (**D**) Quantification of protein expression. Ponceau S staining was utilized as a loading control. Data are presented as relative expression normalized to the NC group. ^#^ *p <* 0.05, ^##^ *p <* 0.01, and ^###^ *p <* 0.01 vs. NC group; ** *p <* 0.01, *** *p <* 0.001 vs. BaCl_2_ group; ^††^ *p* < 0.01 and ^†††^ *p* < 0.001 vs. CIE 100 group; ^‡‡^ *p* < 0.01 and ^‡‡‡^ *p* < 0.001 vs. CIE 200 group. Normal control (NC; phosphate-buffered-saline-treated group); BaCl_2_ (BaCl_2_ + PBS P.O.); and CIE (BaCl_2_ injection + CIE 100, 200, and 400 mg/kg/day P.O., respectively).

The effect of CIE on the skeletal muscle-regeneration-related markers in the TA muscle was analyzed. We investigated MyoG levels in the TA muscle using IHC (Figure 2A). The number of MyoG-positive cells was significantly higher in the BaCl_2_ group compared to the NC group (^###^ *p* < 0.01, 9.6%) (Figure 2B). Treatment with CIE significantly decreased MyoG levels in a dose-dependent manner. The CIE400 group showed a 4.2-fold decrease compared to the BaCl_2_ group, a 4.5-fold decrease compared to the CIE100 group, and a 3.4-fold decrease compared to the CIE200 group, all with statistical significance of *p* < 0.001. MyoG levels after administering 400 mg/kg of CIE were comparable to those in the NC group. Myostatin, a suppressor of satellite cell proliferation and differentiation, maintains muscle homeostasis by preventing excessive muscle growth [24]. Myostatin was significantly downregulated by BaCl_2_ treatment (Figure 2C; ^##^ *p* < 0.01, 68.0%). In contrast, CIE treatment significantly increased myostatin expression in a dose-dependent manner (** *p* < 0.01 and *** *p* < 0.001). Otherwise, differentiation factors, MyoD (^###^ *p* < 0.001, 183.2%) and MyoG (^###^ *p* < 0.001, 69.7%), were significantly upregulated in the BaCl_2_ group compared to the NC group. These myogenic regulatory factors were downregulated dose-dependently, with significant inhibition in the 400 mg/kg CIE group (*** *p* < 0.001 and ** *p* < 0.01, respectively).

### 3.4. CIE Ameliorates Denervation-Induced Muscle Atrophy in TA Muscle

**Figure 3:**
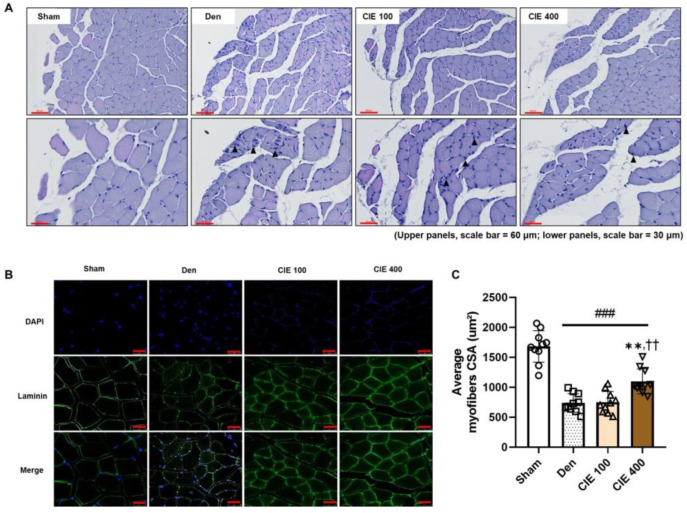
Effects of chestnut inner shell extract (CIE) on histological changes and muscle fiber size in denervated tibialis anterior (TA) muscle. (**A**) Hematoxylin and eosin staining of TA muscle sections revealed morphological changes across different treatment groups. The Sham group displayed normal polygonal-shaped myofibers, while the denervated (Den) group exhibited a reduced fiber size. CIE treatment (100 and 400 mg/kg) demonstrated a dose-dependent improvement in muscle atrophy. Upper panels: 200× magnification (scale bar = 60 μm); Lower panels: 400× magnification (scale bar = 30 μm). (**B**) Immunofluorescence analysis of muscle fiber structure; 4′,6-diamidino-2-phenylindole staining (blue) showed the nuclei, laminin staining (green) marked the basement membrane of muscle fibers, and merged images illustrated the overall muscle structure among different treatment groups. Scale bars = 25 μm. (**C**) Quantification of average myofiber cross-sectional area (CSA) across all groups. The Den group showed significantly reduced CSA compared to Sham (^###^ *p <* 0.001), while CIE 400 mg/kg treatment significantly improved CSA compared to Den and CIE 100 group (** *p <* 0.01 vs. Den; ^††^ *p* < 0.01 vs. CIE 100). Sham (sham control, PBS P.O.); Den (denervation group, PBS P.O.); and CIE (denervation + CIE 100 or 400 mg/kg/day P.O.).

Histological analysis of the TA muscle was conducted to evaluate the morphological changes observed in the Den group compared with those in the Sham group. While the Sham group maintained intact, polygon-shaped myofibers, the Den group exhibited reduced myofiber diameters and centralized nuclei indicative of regeneration (Figure 3A). Treatment with CIE ameliorated these pathological changes in a dose-dependent manner. To quantify the morphological changes in the TA muscle, we conducted IF staining for laminin, which is expressed in the basement membrane of skeletal muscle. The CSA was significantly decreased in the Den group compared to the non-operated group (Figure 3B,C; ^###^ *p* < 0.001, 56.1%). However, 400 mg/kg administration of CIE significantly recovered the morphology (** *p* < 0.01, 34.7%), whereas the 100 mg/kg dose treatment did not lead to a significant improvement compared to the Den group.

### 3.5. CIE Attenuates Denervation-Induced Apoptosis in TA Muscle

**Figure 4:**
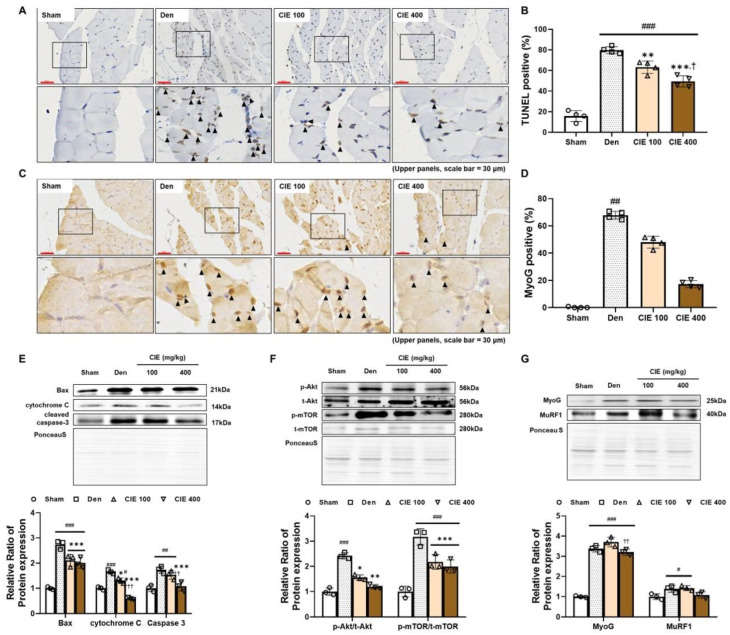
Effects of chestnut inner shell extract (CIE) on atrophy-related factors in denervated (Den) tibialis anterior (TA) muscle. (**A**) The apoptotic cells from Sham, Den, and CIE treatment groups were identified using a terminal deoxynucleotidyl transferase dUTP nick end labeling (TUNEL) assay. The lower panels show magnified views of the boxed regions in the upper images.(**B**) The quantification of TUNEL-positive cells is presented as a percentage. (**C**) Myogenin (MyoG)-positive cells were detected in the Sham, Den, and CIE treatment groups through immunohistochemistry. Magnified images of the boxed areas in the upper panels are shown in the lower panels. (**D**) The quantification of MyoG-positive cells was expressed as a percentage. (**E**) Western blot analysis and quantification of apoptosis-related proteins (Bcl-2-associated X protein (Bax), cytochrome C, cleaved caspase-3) in TA muscle. Full-length blots/gels are located in Figure S2. (**F**) Western blot analysis and quantification of Akt/mTOR pathway proteins (p-protein kinase B (Akt), Akt, p-mammalian target of rapamycin (mTOR), mTOR) in TA muscle. (**G**) Western blot analysis and quantification of MyoG and muscle RING-finger protein-1 (MuRF1) expression in TA muscle. (**F**,**G**) Full-length blots/gels are presented in Figure S3. Data are presented as the mean ± SD. ^#^ *p <* 0.05, ^##^ *p <* 0.01 and ^###^ *p* < 0.001 vs. NC group; * *p <* 0.05, ** *p <* 0.01, and *** *p <* 0.001 vs. BaCl_2_ group; ^†^ *p* < 0.05 and ^††^ *p* < 0.01 vs. CIE 100. Sham (sham control, PBS P.O.); Den (denervation group, PBS P.O.); and CIE (denervation + CIE 100 or 400 mg/kg/day P.O.).

We identified apoptotic cells in the TA muscle using the TUNEL assay (Figure 4A,B). The incidence of TUNEL positivity was much higher due to denervation compared to the Sham group (^###^ *p* < 0.001, 64.0%). Conversely, CIE administration significantly lowered this ratio in a dose-dependent manner (** *p* < 0.01 and *** *p* < 0.001). We also performed immunostaining for MyoG in the TA muscle (Figure 4C,D). MyoG positivity was observed in the affected regions, characterized by a smaller myofiber diameter in the denervation-operated groups. The number of MyoG-positive cells per area showed a significant rise in the Den group compared to the Sham group (^##^ *p* < 0.01, 65.3%). The administration of CIE significantly reduced positivity in a dose-dependent manner (ns).

Immunoblotting was performed to investigate the mechanism of apoptosis in the TA muscle. The apoptotic markers Bax, cytochrome C, and cleaved caspase-3 were significantly increased by denervation (Figure 4E; ^###^ *p* < 0.001; 181.2, 79.8, and 81.3%, respectively). However, a significant dose-dependent decrease in these protein levels was found in the CIE treatment groups (*** *p* < 0.001). Additionally, the activation of Akt and mTOR was significantly increased by sciatic nerve dissection (Figure 4F; ^###^ *p* < 0.001; 128.6 and 223.5%, respectively). In contrast, CIE treatment significantly reduced these expression levels in a concentration-dependent manner (* *p* < 0.05 and ** *p* < 0.01 in Akt; *** *p* < 0.001 in mTOR). The expression of MyoG, which is activated by denervation-induced muscle atrophy [25], was significantly increased with denervation (Figure 4G; ^###^ *p* < 0.001, 237.7%); 400 mg/kg administration of CIE downregulated its expression by 1.2-fold compared to CIE 100 (^††^ *p* < 0.01). The protein expression of MuRF1 was significantly higher in the Den group (^#^ *p* < 0.05, 37.9%). The administration of 400 mg/kg of CIE markedly reduced the expression level of MuRF1 compared to the Den group (ns).

### 3.6. CIE Attenuates Oxidative Stress from H_2_O_2_ in C2C12 Myoblasts

**Figure 5:**
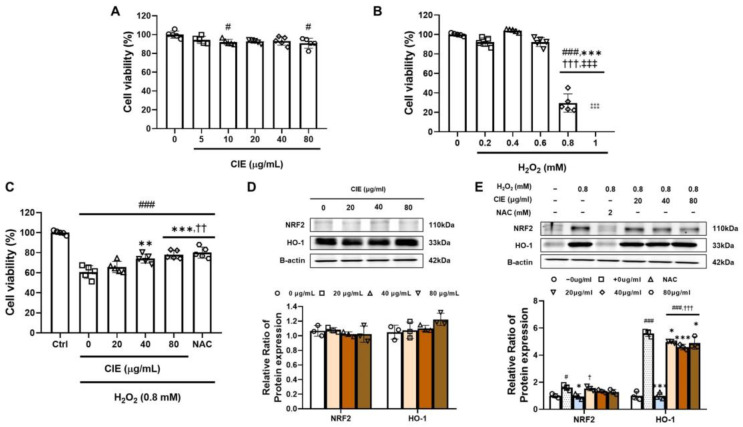
Effect of chestnut inner shell extract (CIE) on C2C12 myoblasts under oxidative stress. (**A**) Evaluation of cell viability in C2C12 myoblasts subjected to a range of CIE concentrations (0–80 μg/mL) for 24 h. ^#^ *p <* 0.05 vs. 0. (**B**) Assessment of cell viability in C2C12 myoblasts that were exposed to different concentrations of hydrogen peroxide (H_2_O_2_) (0–1 mM) for 24 h. ^###^ *p <* 0.001 vs. 0; *** *p <* 0.001 vs. 0.2; ^†††^ *p* < 0.001 vs. 0.4; ^‡‡‡^ *p* < 0.001 vs. 0.6; ^⁑⁑⁑^ *p* < 0.001 vs. 0.8 mM of H_2_O_2_. (**C**) Protective effects of CIE (0–80 μg/mL) against oxidative stress induced by H_2_O_2_ (0.8 mM) on the cell viability of C2C12 myoblasts. ^###^ *p <* 0.001 vs. Ctrl; ** *p <* 0.01 and *** *p <* 0.001 vs. 0; ^††^ *p* < 0.01 vs. 20 μg/mL of CIE. (**D**) Examination of the expression of nuclear factor erythroid 2 (NRF2) and heme oxygenase 1 (HO-1) proteins in C2C12 myoblasts treated with different concentrations of CIE without H_2_O_2_ exposure. Full-length blots/gels are presented in Figure S4. (**E**) Analysis of NRF2 and HO-1 expression levels in C2C12 myoblasts treated with H_2_O_2_ (0.8 mM) and N-acetyl cysteine (NAC) or various concentrations of CIE. Full-length blots/gels are presented in Figure S5. ^#^ *p <* 0.05 and ^###^ *p <* 0.001 vs. −0; * *p <* 0.05 and *** *p <* 0.001 vs. +0; ^†^ *p* < 0.05 and ^†††^ *p* < 0.001 vs. NAC. Data are presented as the mean ± SD.

Before assessing the impact of CIE on myoblasts, we treated C2C12 cells with H_2_O_2_ to induce oxidative stress, which has been reported as a common overarching mechanism in two in vivo experiments [8]. We found that CIE was non-toxic to C2C12 cells at concentrations up to 80 μg/mL after 24 h of co-treatment (Figure 5A). H_2_O_2_ preserved cell viability at concentrations up to 0.6 mM; however, there was a significant drop in viability at 0.8 mM (^###^ *p* < 0.001, 58.9%) (Figure 5B). Therefore, we selected this concentration to induce oxidative-stress-related muscle damage in C2C12 myoblasts. The protective effects of CIE on C2C12 myoblasts were confirmed through treatment with H_2_O_2_ (Figure 5C). The application of H_2_O_2_ significantly decreased cell viability compared to the control group (^###^ *p* < 0.001, 41.1%). The antioxidant NAC notably enhanced cell viability (*** *p* < 0.001). CIE treatment significantly reduced oxidative stress toxicity in a dose-dependent manner (** *p* < 0.01 and *** *p* < 0.001). To evaluate CIE’s antioxidative effects on C2C12 myoblasts, we treated the cells with CIE or without H_2_O_2_. The administration of CIE did not cause a significant rise in NRF2 and HO-1 expression in the absence of H_2_O_2_ (Figure 5D). After inducing oxidative stress in C2C12 myoblasts, NRF2, which responds to such stress, showed a significant increase (Figure 5E; ^#^ *p* < 0.05, 59.9%). NAC treatment effectively restored the expression back to levels similar to the control (* *p* < 0.05). CIE treatment downregulated NRF2 expression in a concentration-dependent manner (ns). The expression of HO-1, an antioxidant enzyme downstream of NRF2, showed a similar pattern. The induction of oxidative stress significantly upregulated its expression (^###^ *p* < 0.001, 460.2%). NAC, the positive control, significantly reduced the expression to levels comparable to the control (*** *p* < 0.001). Treatment with CIE significantly prevent the upregulation of NRF2 and HO-1 (* *p* < 0.05 and *** *p* < 0.001).

## 4. Discussion

In this study, we discovered that orally administered CIE has a pharmaceutical effect on acute skeletal muscle injury and atrophy using two in vivo models. CIE treatment reduced BaCl_2_-induced damage to the TA muscle and protected against muscle atrophy induced by nerve dissection. Moreover, CIE alleviated oxidative stress damage in C2C12 myoblasts.

We investigated the effect of CIE on muscle damage in a mouse model of BaCl_2_-induced muscle injury. BaCl_2_ injection into the skeletal muscle leads to necrosis, followed by compensatory regeneration that includes inflammation with monocyte infiltration, the proliferation and differentiation of myoblasts, and myotube formation [4,26]. In our model, newly formed myofibers with central nuclei were observed 10 days after BaCl_2_ injection, consistent with previous studies reporting that regeneration begins within a few days post-injury by 10 days [27,28,29]. In the grip strength test, the muscle strength was higher in the CIE-treated group. Moreover, CIE administration decreased the ratio of centrally nucleated myofibers with an increased myofiber diameter, similar to the NC. Therefore, our findings suggest that the oral administration of CIE protects skeletal muscles from BaCl_2_-induced damage.

We also assessed the effectiveness of CIE in a mouse model of muscle atrophy caused by denervation. When neuronal stimuli are lost due to injury or degeneration, muscle fibers accumulate oxidative stress and undergo apoptosis, leading to muscle sarcopenia [30]. Four weeks after the surgery, we observed hallmark features of muscle atrophy, including a reduced myofiber cross-sectional area and centralized pyknotic nuclei [31] CIE administration mitigated these changes in a dose-dependent manner, as evidenced by the preserved muscle weight and fiber CSA. Our results may hold therapeutic promise as an adjunctive treatment.

Myogenic regulators control muscle regeneration. Under normal conditions, satellite cells are maintained in quiescence by growth-regulating signaling molecules, such as myostatin. Upon muscle injury, these cells become activated, proliferate, and differentiate into MyoD-expressing myoblasts, which subsequently fuse into multinucleated myofibers expressing MyoG after MyoD downregulation, and finally mature into myofibers as MyoD and MyoG expression declines [32]. In our BaCl_2_-induced muscle injury model, the expression of MyoD and MyoG was markedly elevated in the BaCl_2_ group but was downregulated in a dose-dependent manner following CIE administration. This trend is consistent with the improved functional and morphological recovery, including restored grip strength, reduced proportions of centrally nucleated fibers, and normalized myofiber diameter in the high-dose CIE group. The decreased expression of MyoD and MyoG likely reflects a transition from an active state of regeneration to a more quiescent, mature muscle state. This interpretation is supported by the near absence of MyoG-positive staining in immunohistochemistry and the histological similarity of myofibers to the non-injured control group. Although myostatin is widely recognized as a negative regulator of muscle growth, it is also essential for maintaining muscle homeostasis. Myostatin expression was significantly decreased in the BaCl_2_-injured muscle but was restored by CIE treatment to levels comparable to the non-injured group. Rather than inhibiting regeneration, this normalization may indicate the completion of regenerative processes and a reestablishment of the physiological balance [28,33].

The loss of neuronal stimuli in skeletal muscle increases oxidative stress, leading to mitochondrial damage, which causes myofiber apoptosis and results in denervation-induced muscle atrophy [34]. Previous studies have shown that denervation not only induces the upregulation of MuRF1 and atrogin-1, which contribute to muscle atrophy by promoting protein degradation, but also enhances the activation of the forkhead box O and Akt/mTOR signaling pathways, which trigger apoptosis [35,36]. Our results showed that CIE administration in a dose-dependent manner inhibited MuRF1, atrogin-1, and forkhead box O/Akt/mTOR signaling in the denervation model, which was evidenced by decreased cleaved caspase-3 and TUNEL staining. Based on these results, the previously observed histological protective effects of CIE against muscle damage and atrophy were supported by changes in the expression of regenerative mechanisms and apoptosis-related proteins that induce muscle atrophy in response to muscle damage.

Both BaCl_2_-injection- and sciatic-nerve-transection-induced denervation have been reported to be correlated with oxidative stress [37,38]. Oxidative stress is a crucial contributor to skeletal muscle atrophy, inflammation, and mitochondrial dysfunction, as it damages cells through lipid peroxidation and DNA damage [39,40]. Upon the deprivation of neuronal stimulation induced by mechanical loading, the antioxidative capacity of mitochondria is reduced, leading to the accumulation of ROS, which then initiates the apoptosis of skeletal muscle [41]. In our in vitro study, CIE administration significantly protected cells from H_2_O_2_-induced oxidative damage. H_2_O_2_ treatment markedly upregulated NRF2 and HO-1 expression, reflecting a compensatory antioxidant response. However, both NAC—a well-known antioxidant—and CIE treatment significantly suppressed this upregulation in a dose-dependent manner. These findings suggest that CIE, like NAC, can diminish NRF2/HO-1 activity by alleviating oxidative stress [42]. These findings support the potential protection of CIE against muscle injury and atrophy by relieving oxidative stress.

## 5. Conclusions

In conclusion, CIE protects against muscle damage from BaCl_2_ and inhibits muscle atrophy in denervation models. This was confirmed using an H_2_O_2_-induced muscle damage model in C2C12 cells, where CIE reduced oxidative stress, inhibiting muscle damage and atrophy. These findings suggest that CIE has pharmaceutical potential for treating skeletal muscle myopathies and could serve as a novel supplemental agent.

## Figures and Tables

**Table 1:** Effects of chestnut inner shell extract (CIE) on the body weight (BW) and the ratio of injured tibialis anterior (TA) to intact muscle weight in mice with barium chloride (BaCl_2_)-induced muscle injury.

	NC	BaCl_2_	CIE 100	CIE 200	CIE 400
BW before CIE treatment (g)	22.0 ± 0.4	21.8 ± 0.3	22.6 ± 0.6	21.8 ± 0.5	22.1 ± 0.6
BW 15 days after CIE treatment (g)	26.2 ± 0.8	25.0 ± 0.6	26.1 ± 1.1	24.7 ± 0.8	25.7 ± 1.9
Injured TA weight (mg)	55.6 ± 7.6	52.8 ± 5.9	52.0 ± 10.7	53.2 ± 5.7	54.6 ± 9.2
Injured/non-injured TA weight	1.2 ± 0.3	0.9 ± 0.1 ^#^	0.9 ± 0.1	1.0 ± 0.1	1.1 ± 0.3

Data are presented as the mean ± SD (*n* = 6 per group). ^#^ *p* < 0.05 vs. NC group. Normal control (NC; phosphate-buffered saline-treated group); BaCl_2_ (BaCl_2_ + PBS P.O.); and CIE (BaCl_2_ injection + CIE 100, 200, and 400 mg/kg/day P.O., respectively).

**Table 2:** Effects of chestnut inner shell extract (CIE) on body weight (BW) and the comparison of the ratio of denervated (Den) tibialis anterior (TA) to the weight of non-denervated muscle in mice.

	Sham	Den	CIE 100	CIE 400
BW before surgery (g)	20.7 ± 1.2	21.4 ± 1.0	21.1 ± 0.4	20.8 ± 0.9
BW 1 week after surgery (g)	21.4 ± 1.2	21.2 ± 0.9	20.7 ± 0.6	21.8 ± 0.7
BW 2 week after surgery (g)	21.8 ± 0.8	22.0 ± 1.3	21.5 ± 1.7	22.6 ± 1.1
BW 3 week after surgery (g)	24.1 ± 1.5	23.9 ± 1.2	22.9 ± 1.4	23.6 ± 1.3
BW 4 week after surgery (g)	23.8 ± 1.3	23.4 ± 1.2	23.2 ± 1.0	23.6 ± 1.3
Denervated TA weight (mg)	39.7 ± 3.5	28.4 ± 5.0	33.7 ± 2.8	33.0 ± 5.1
Denervated/non-denervated TA weight	1.0 ± 0.1	0.7 ± 0.1 ^##^	0.6 ± 0.1	0.9 ± 0.1 *

Data are presented as the mean ± SD (*n* = 6 per group). ^##^ *p* < 0.01 vs. NC group. * *p* < 0.05 vs. Den group. Sham (sham control, PBS P.O.); Den (denervation group, PBS P.O.); and CIE (denervation + CIE 100 or 400 mg/kg/day P.O.).

## Data Availability

The original contributions presented in this study are included in the article and Supplementary Materials. Further inquiries can be directed to the corresponding authors.

## Supplementary Materials

The following supporting information can be downloaded at: https://www.mdpi.com/article/10.3390/nu17132116/s1, Figure S1: Full-length blots/gels of Figure 2C; Figure S2: Full-length blots/gels of Figure 4E; Figure S3: Full-length blots/gels of Figure 4F,G; Figure S4: Full-length blots/gels of Figure 5D; Figure S5: Full-length blots/gels of Figure 5E.

## Abbreviations

The following abbreviations are used in this manuscript:

AKT	Protein kinase B
BaCl_2_	Barium chloride
Bax	Bcl-2 associated X protein
CIE	Chestnut inner shell extract
CSA	Cross-sectional area
Den	Denervation operated group
DMEM	Dulbecco’s modified eagle medium
DMSO	Dimethyl sulfoxide
H&E	Hematoxylin and eosin
H_2_O_2_	Hydrogen peroxide
HO-1	Heme oxygenase 1
IF	Immunofluorescence
IHC	Immunohistochemistry
mTOR	Mammalian target of rapamycin
MuRF1	Muscle RING-finger protein-1
MyoD	Myogenic differentiation 1
MyoG	Myogenin
NAC	N-acetyl-L-cysteine
NC	Normal control
NRF2	Nuclear factor erythroid 2
PBS	Phosphate-buffered saline
PO	Per os
PVDF	Polyvinylidene difluoride
RIPA	Radio immunoprecipitation assay
ROS	Reactive oxygen species
SD	Standard deviation
TA	Tibialis anterior
TUNEL	Terminal deoxynucleotidyl transferase dUTP nick end labeling

## References

[1] Carlson B. (2021). Muscle disorders. Muscle Biology.

[2] Olivé M., Ferrer I. (2000). Bcl-2 and bax immunohistochemistry in denervation-reinnervation and necrosis-regeneration of rat skeletal muscles. Muscle Nerve.

[3] Fleming J.W., Capel A.J., Rimington R.P., Wheeler P., Leonard A.N., Bishop N.C., Davies O.G., Lewis M.P. (2020). Bioengineered human skeletal muscle capable of functional regeneration. BMC Biol..

[4] Morton A.B., Norton C.E., Jacobsen N.L., Fernando C.A., Cornelison D.D.W., Segal S.S. (2019). Barium chloride injures myofibers through calcium-induced proteolysis with fragmentation of motor nerves and microvessels. Skelet. Muscle.

[5] Matuz-Mares D., González-Andrade M., Araiza-Villanueva M.G., Vilchis-Landeros M.M., Vázquez-Meza H. (2022). Mitochondrial calcium: Effects of its imbalance in disease. Antioxidants.

[6] Xie W.Q., He M., Yu D.J., Wu Y.X., Wang X.H., Lv S., Xiao W.F., Li Y.S. (2021). Mouse models of sarcopenia: Classification and evaluation. J. Cachexia Sarcopenia Muscle.

[7] Scalabrin M., Pollock N., Staunton C.A., Brooks S.V., McArdle A., Jackson M.J., Vasilaki A. (2019). Redox responses in skeletal muscle following denervation. Redox. Biol..

[8] Hwangbo H., Park C., Bang E., Kim H.S., Bae S.J., Kim E., Jung Y., Leem S.H., Seo Y.R., Kim G.Y. (2024). Morroniside protects C2C12 myoblasts from oxidative damage caused by ROS-mediated mitochondrial damage and induction of endoplasmic reticulum stress. Biomol. Ther..

[9] Mankhong S., Kim S., Moon S., Kwak H.B., Park D.H., Kang J.H. (2020). Experimental models of sarcopenia: Bridging molecular mechanism and therapeutic strategy. Cells.

[10] Chun S.E., Lee S.H., Shin Y.J., Shin S.H. (2023). Herbal medicine for sarcopenia: A systematic review of randomized controlled trials. J. Int. Korean Med..

[11] Saeed M., Tasleem M., Haque A., Shoaib A., Rizvi S.M.D. (2025). Muscular dystrophies and therapeutic potential of medicinal plants. JDR.

[12] Bagherniya M., Mahdavi A., Shokri-Mashhadi N., Banach M., Von Haehling S., Johnston T.P., Sahebkar A. (2022). The beneficial therapeutic effects of plant-derived natural products for the treatment of sarcopenia. J. Cachexia Sarcopenia Muscle.

[13] Jeong J.S., Kim J.W., Kim J.H., Kim C.Y., Ko J.W., Kim T.W. (2023). Protective effects of chestnut (*Castanea crenata*) inner shell extract in macrophage-driven emphysematous lesion induced by cigarette smoke condensate. Nutrients.

[14] Frati A., Landi D., Marinelli C., Gianni G., Fontana L., Migliorini M., Pierucci F., Garcia-Gil M., Meacci E. (2014). Nutraceutical properties of chestnut flours: Beneficial effects on skeletal muscle atrophy. Food Funct..

[15] Tuyen P.T., Xuan T.D., Khang D.T., Ahmad A., Quan N.V., Tu Anh T.T., Anh L.H., Minh T.N. (2017). Phenolic Compositions and Antioxidant Properties in Bark, Flower, Inner Skin, Kernel and Leaf Extracts of Castanea crenata Sieb. et Zucc. Antioxidants.

[16] Liu X., Cheng C., Deng B., Liu M. (2022). Ellagic acid attenuates muscle atrophy in STZ-induced diabetic mice. Physiol. Res..

[17] Yu L., Tian D., Su Z., Zhang L., Guo S., Zhu W., Fang Y., Wang P., Zhang N. (2025). Gallic acid alleviates exercise-induced muscle damage by inhibiting mitochondrial oxidative stress and ferroptosis. J. Transl. Med..

[18] Jeong J.S., Kim J.W., Kim J.H., Kim C.Y., Ko J.W., Kim T.W. (2024). Korean red ginseng suppresses mitochondrial apoptotic pathway in denervation-induced skeletal muscle atrophy. J. Ginseng Res..

[19] Zheng Y., Lunn A., Gao J., Chen H., Yao Y. (2024). Quantitative evaluation of hindlimb grip strength in mice as a measure of neuromuscular function. MethodsX.

[20] El-Habta R., Andersson G., Kingham P.J., Backman L.J. (2021). Anti-apoptotic effect of adipose tissue-derived stromal vascular fraction in denervated rat muscle. Stem. Cell Res. Ther..

[21] Fortes M.A.S., Marzuca-Nassr G.N., Vitzel K.F., da Justa Pinheiro C.H., Newsholme P., Curi R. (2016). Housekeeping proteins: How useful are they in skeletal muscle diabetes studies and muscle hypertrophy models?. Anal. Biochem..

[22] Wang Q., Han W., Ma C., Wang T., Zhong J. (2023). Western blot normalization: Time to choose a proper loading control seriously. Electrophoresis.

[23] Meyer G.A. (2018). Evidence of induced muscle regeneration persists for years in the mouse. Muscle Nerve.

[24] Kostyunina D.S., Ivanova A.D., Smirnova O.V. (2018). Myostatin: Twenty years later. Hum. Physiol..

[25] Macpherson P.C.D., Wang X., Goldman D. (2011). Myogenin regulates denervation-dependent muscle atrophy in mouse soleus muscle. J. Cell Biochem..

[26] Dekeyser G.J., Clary C.R., Otis J.S. (2013). Chronic alcohol ingestion delays skeletal muscle regeneration following injury. Regen Med. Res..

[27] Musarò A. (2014). The basis of muscle regeneration. Adv. Biol..

[28] Forcina L., Cosentino M., Musarò A. (2020). Mechanisms Regulating Muscle Regeneration: Insights into the Interrelated and Time-Dependent Phases of Tissue Healing. Cells.

[29] Jung H.W., Choi J.H., Jo T., Shin H., Suh J.M. (2019). Systemic and local phenotypes of barium chloride induced skeletal muscle injury in mice. Ann. Geriatr. Med. Res..

[30] Cisterna B.A., Cardozo C., Sáez J.C. (2014). Neuronal involvement in muscular atrophy. Front. Cell Neurosci..

[31] Wang B.B., Guo C., Sun S.Q., Zhang X.N., Li Z., Li W.J., Li D.Z., Schumacher M., Liu S. (2023). Comparison of the nerve regeneration capacity and characteristics between sciatic nerve crush and transection injury models in rats. Biomed. Env. Sci..

[32] Schmidt M., Schüler S.C., Hüttner S.S., von Eyss B., von Maltzahn J. (2019). Adult stem cells at work: Regenerating skeletal muscle. Cell Mol. Life Sci..

[33] Mendler L., Zádor E., Ver Heyen M., Dux L., Wuytack F. (2000). Myostatin levels in regenerating rat muscles and in myogenic cell cultures. J. Muscle Res. Cell Motil..

[34] Adhihetty P.J., O’Leary M.F.N., Chabi B., Wicks K.L., Hood D.A. (2007). Effect of denervation on mitochondrially mediated apoptosis in skeletal muscle. J. Appl. Physiol..

[35] MacDonald E.M., Andres-Mateos E., Mejias R., Simmers J.L., Mi R., Park J.S., Ying S., Hoke A., Lee S.J., Cohn R.D. (2014). Denervation atrophy is independent from Akt and mTOR activation and is not rescued by myostatin inhibition. Dis. Model Mech..

[36] Zhang X., Tang N., Hadden T.J., Rishi A.K. (2011). Akt, FoxO, and regulation of apoptosis. Biochim. Biophys. Acta. Mol. Cell Res..

[37] Elwej A., Chaabane M., Ghorbel I., Chelly S., Boudawara T., Zeghal N. (2017). Effects of barium graded doses on redox status, membrane bound ATPases and histomorphological aspect of the liver in adult rats. Toxicol. Mech. Methods.

[38] Powers S.K. (2014). Can antioxidants protect against disuse muscle atrophy?. Sports Med..

[39] Shields H.J., Traa A., Van Raamsdonk J.M. (2021). Beneficial and detrimental effects of reactive oxygen species on lifespan: A comprehensive review of comparative and experimental studies. Front. Cell. Dev. Biol..

[40] Zhang H., Qi G., Wang K., Yang J., Shen Y., Yang X., Chen X., Yao X., Gu X., Qi L. (2023). Oxidative stress: Roles in skeletal muscle atrophy. Biochem. Pharmacol..

[41] Ji L.L., Yeo D. (2019). Mitochondrial dysregulation and muscle disuse atrophy. F1000Res.

[42] Lee D., Kook S.H., Ji H., Lee S.A., Choi K.C., Lee K.Y., Lee J.C. (2015). N-acetyl cysteine inhibits H_2_O_2_-mediated reduction in the mineralization of MC3T3-E1 cells by down-regulating Nrf2/HO-1 pathway. BMB Rep..

