# Supplementary material for: Chestnut (Castanea crenata) Inner-Shell Extract Attenuates Barium-Chloride-Induced Injury and Denervation-Induced Atrophy in Skeletal Muscle of Mice
Source: Nutrients. 2025 Jun 26;17(13):2116. doi: 10.3390/nu17132116 (PMC12251227; doi:10.3390/nu17132116)

Supplementary Figure S1. Full-length blots/gels of Figure 2C.

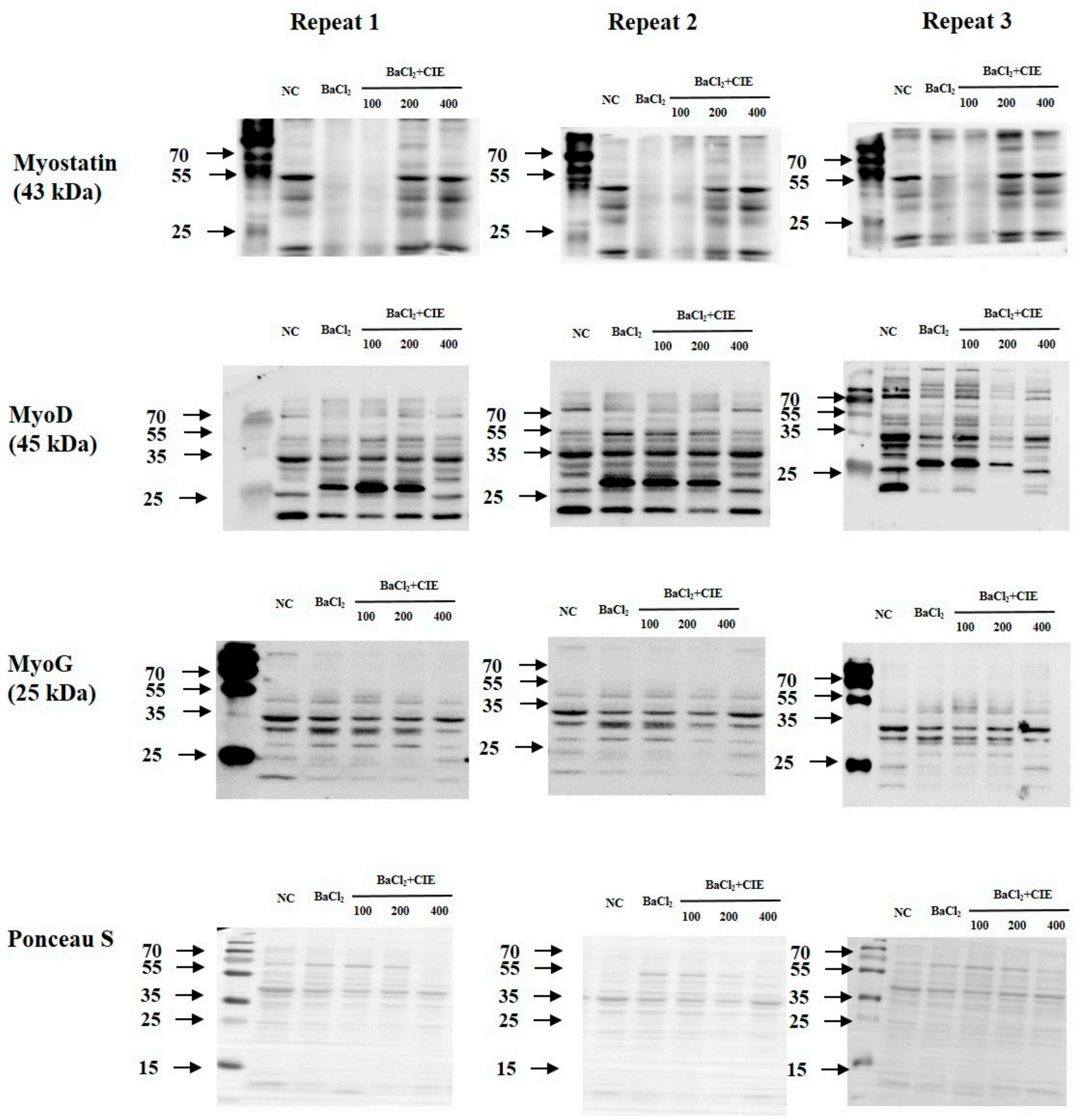

Supplementary Figure S2. Full-length blots/gels of Figure 4E.

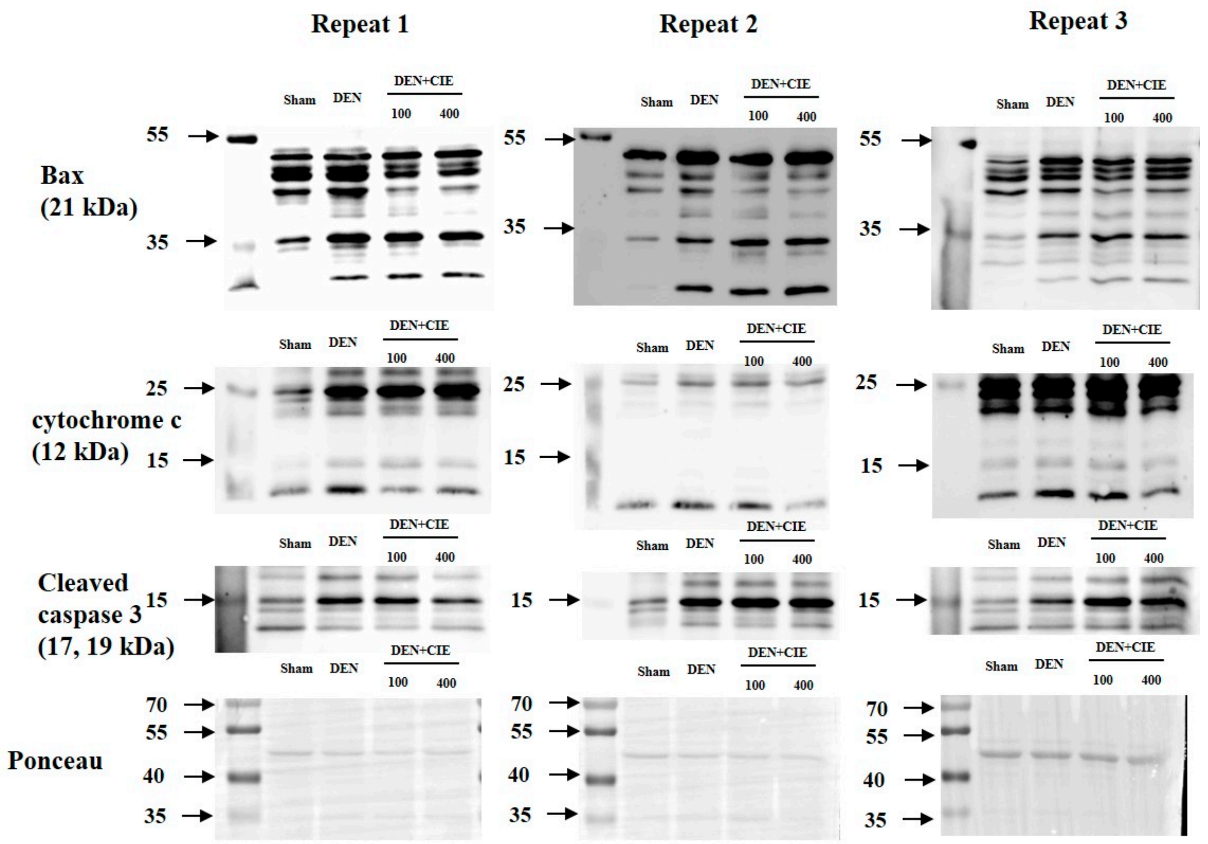

Supplementary Figure S3. Full-length blots/gels of Figure 4F,G.

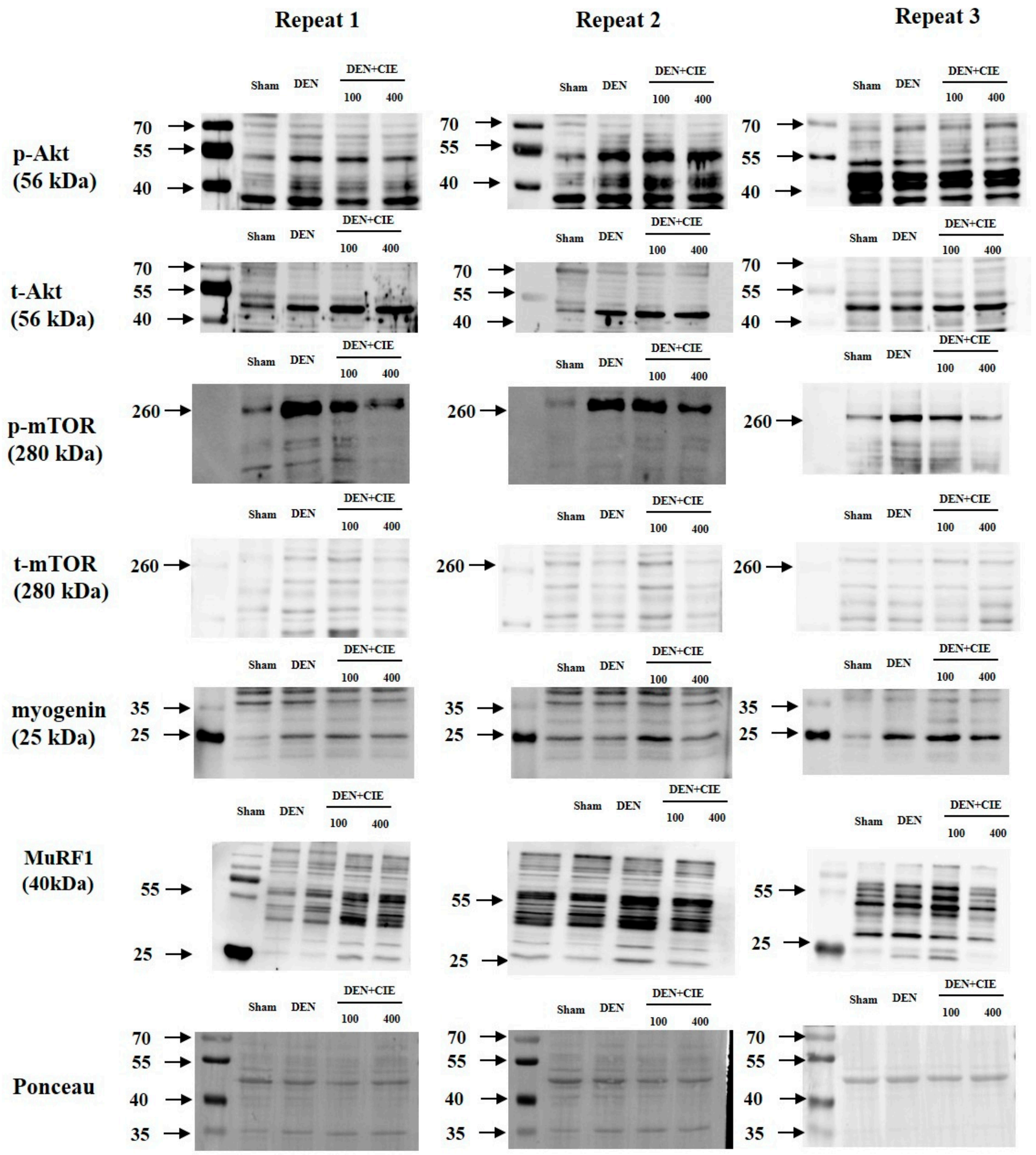

Supplementary Figure S4. Full-length blots/gels of Figure 5D.

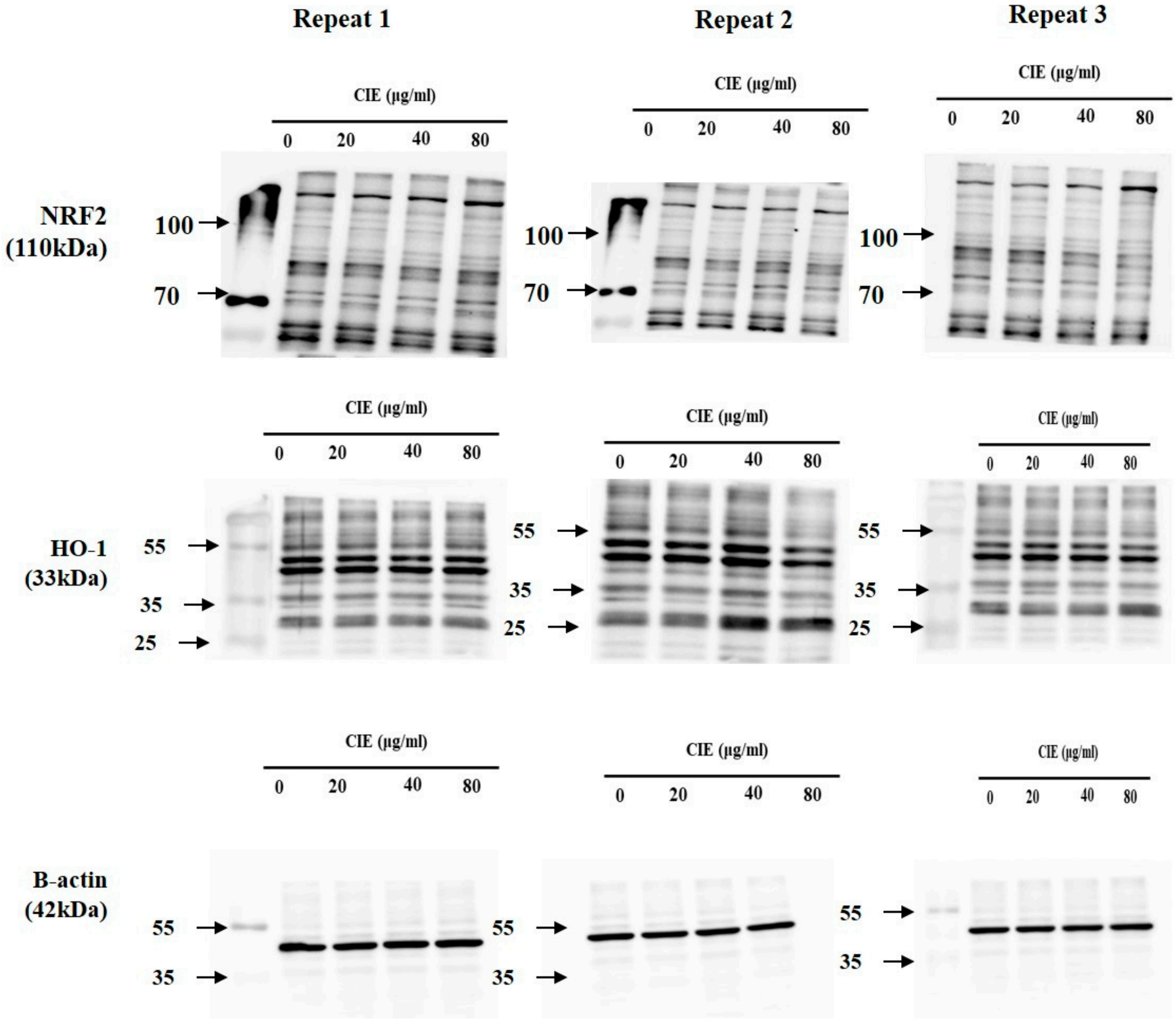

Supplementary Figure S5. Full-length blots/gels of Figure 5E.

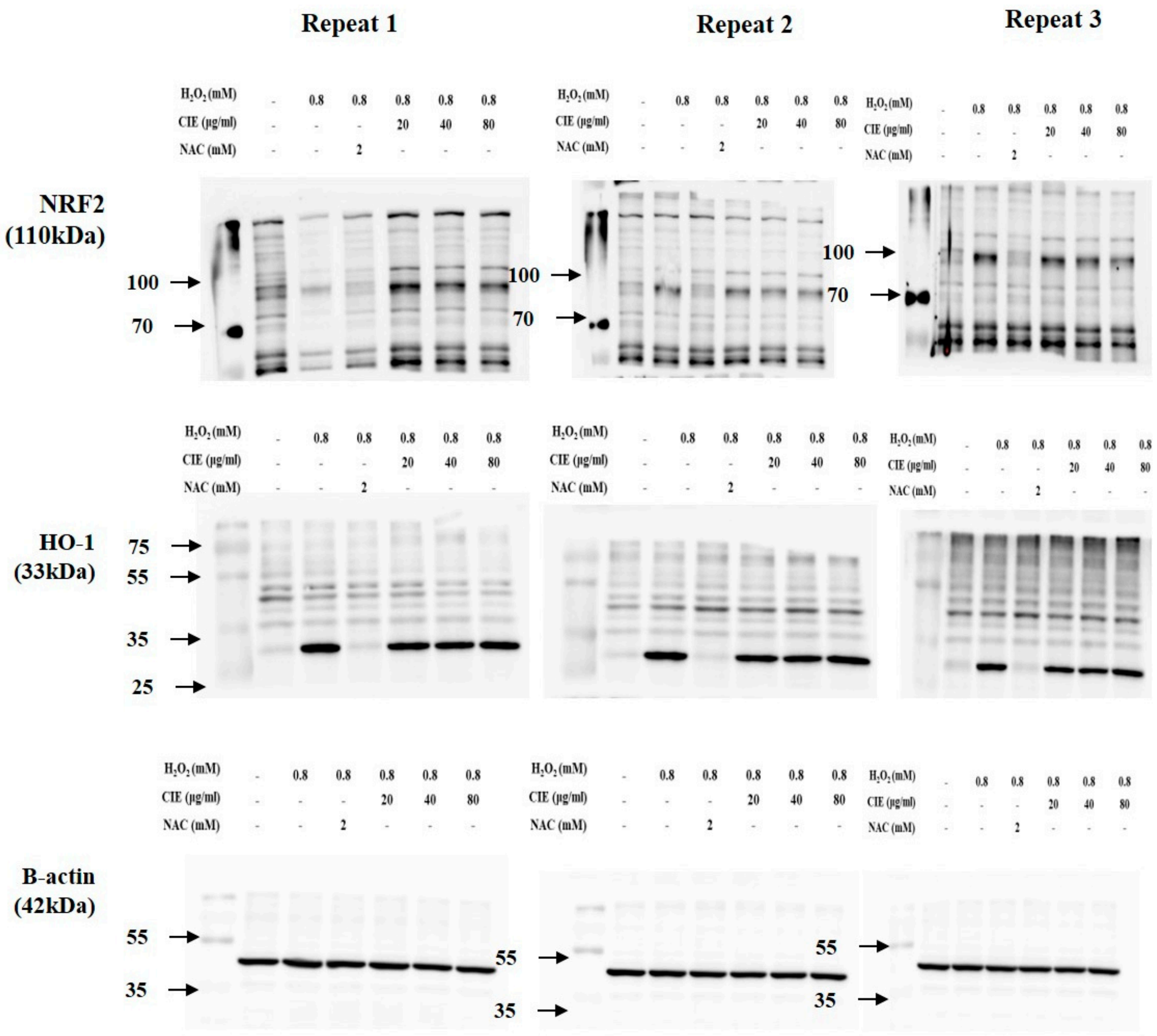

Supplement: Supplementary file 1 [file nutrients-17-02116-s001.zip › nutrients-3666351-supplementary.pdf]
